# Comparative Analysis of miRNA Expression 106a‐5p and 375‐3p and Proteins ERK1/2, p38, β‐Catenin and E‐Cadherin in Prostate Cancer and Benign Prostatic Hyperplasia

**DOI:** 10.1111/jcmm.70695

**Published:** 2025-08-21

**Authors:** Magdalena Smereczańska, Natalia Domian, Grzegorz Młynarczyk, Irena Kasacka

**Affiliations:** ^1^ Department of Histology and Cytophysiology Medical University of Bialystok Bialystok Poland; ^2^ Department of Urology Medical University of Bialystok Bialystok Poland

**Keywords:** E‐cadherin, ERK1/2, microRNA, p38, prostate cancer, β‐catenin

## Abstract

Prostate cancer is the most frequently diagnosed cancer in men and the second most common cause of death. PSA is performed in men to detect prostate cancer early, but PSA levels increase in both cancer and BPH. Therefore, there is an important need to understand prostate‐specific molecular signatures. Considering the importance of the MAPK and Wnt/β‐catenin pathways in the progression of many cancers and the fact that the miRNA expression profile is unique in each cancer type, evaluation of 106a‐5p and 375‐3p miRNAs may be an important factor in cancer detection. The research was carried out on tissue fragments taken from 30 patients with BPH and 30 with cancer. IHC and qRT‐PCR were used to identify the expression of tested proteins, while the expression of miRNAs was examined using dPCR. The results of the present study showed significantly higher fluorescence intensity of miRNAs 106a‐5p and 375‐3p, and lower immunoreactivity and the expression of genes encoding ERK1/2, p38, β‐catenin and E‐cadherin in prostate cancer compared to BPH. It is possible that thanks to miRNA 106a‐5p and 375‐3p, the proteins we studied belonging to the MAPK and Wnt/β‐catenin pathways play a protective suppressor role in prostate cancer cells.

**Abbreviations**
BPH: benign prostatic hyperplasia
dPCR: digital PCR
MAPK: Mitogen‐activated protein kinase
miRNAs: microRNAs
PSA: prostate‐specific antigen

## 1 Introduction

Benign prostatic hyperplasia (BPH) is characterised by enlargement of the prostate gland due to numerous divisions of prostate cells and stromal elements [1, 2]. Whereas prostate cancer (prostate adenocarcinoma) is the most common cancer in men over 50, which arises from the peripheral zone of the prostate gland. In the initial phase of cancer, symptoms, such as urge to urinate and frequent urination are similar to BPH, while in the phase of invasion of adjacent tissues and metastases to other organs, most often to bones, pain occurs. That is why correct urological diagnosis is so important, because low‐grade prostate cancer does not require treatment, but only monitoring of the lesion, while in the case of a malignant form, treatment is required [3].

The diagnosis of prostate cancer is mainly based on the examination of prostate‐specific antigen (PSA) produced by prostate glandular epithelial cells [4]. Unfortunately, PSA levels increase not only in cancer but also in BPH, so it is extremely important to find specific molecular signatures to distinguish benign hyperplasia from cancer [5].

MicroRNAs (miRNAs) are short, non‐coding, single‐stranded molecules involved in the regulation of gene expression at the post‐transcriptional level by binding to mRNA [6]. Research on new miRNAs and the detection of changes in their expression may contribute to the early detection of cancer cells and serve as a prognostic factor for the course and treatment of the disease. Defining target genes for dysregulated miRNAs in cancer cells and developing methods for their selective silencing is a promising therapeutic strategy [7, 8].

Abnormal miRNA levels may involve various stages of cancer pathogenesis, such as apoptosis, proliferation, or metastasis. In various novel studies conducted on prostate tissues and prostate cancer cell lines, it was shown that there are various miRNA signatures acting as suppressor genes, for example, miR‐211, miR‐202, and miR‐139 [1, 9, 10, 11]. During the transformation of normal cells, oncogenic miRNAs are often overexpressed, while tumour‐suppressive miRNAs are downregulated. Since each type of cancer has a distinct miRNA expression profile, numerous studies have attempted to assess the clinical significance of miRNAs as diagnostic tools [12, 13].

Studies conducted in recent years have shown that selected miRNAs regulate the proliferation and migration of cancer cells by modulating the MAPK and Wnt/β‐catenin pathways. A close relationship has been demonstrated between the expression of proteins belonging to the MAPK (ERK1/2, p38) and Wnt (β‐catenin, E‐cadherin) pathways and the degree of tumour differentiation, the presence of metastases, and survival. One of the novel studies showed that by modulating the MAPK pathway, miR‐106a exerts antiproliferative effects on bladder cancer cells [14]. Moreover, miR‐375 inhibits the growth of cancer cells, stopping them in the G0 phase [15].

In many urological cancers, ERK1/2, p38, β‐catenin, and E‐cadherin are involved in tumour transformation through the RAS/RAF/MEK/ERK or Wnt/β‐catenin pathways. Literature data indicate the success of the first therapies using MAPK pathway inhibitors in the treatment of cancer by inhibiting ERK activity or inhibiting the phosphorylation process of this kinase. Considering the importance of the MAPK pathway in the progression of many cancers and the fact that the miRNA expression profile is unique in each type of cancer, the assessment of selected miRNAs may be an invaluable method for detecting cancer cells and act as a prognostic factor in the development of the disease [5, 16, 17, 18].

Despite numerous studies carried out so far in the field of oncology, our knowledge of cellular signalling networks—miRNA in the process of carcinogenesis is still insufficient [14]. This inspired the search for miRNAs that could be used to differentiate prostate cancer from BPH in men with elevated PSA levels. Therefore, we hypothesize that the protein expression levels of ERK1/2, p38, β‐catenin, and E‐cadherin, as well as miRNAs 106a‐5p and 375‐3p, differ significantly between prostate cancer and BPH tissues, and that these molecular differences may reflect the involvement of MAPK and Wnt/β‐catenin signalling pathways in prostate cancer progression. Furthermore, the altered expression of these miRNAs may regulate these pathways and serve as potential molecular markers to distinguish prostate cancer from BPH.

Thus, the aim of our research was to verify whether selected miRNAs have an effect on modulating elements of the MAPK and Wnt/β‐catenin pathways in prostate cancer compared to BPH. The expression of 106a‐5p and 375‐3p miRNAs was analysed in tissues by Digital PCR. Additionally, immunohistochemical and molecular evaluation of ERK1/2, p38, β‐catenin, and E‐cadherin in prostate cancer compared to BPH was performed to better understand their role in oncogenesis.

## 2 Material & Methods

### 2.1 Sample Collection

The research was carried out on postoperative material collected from 30 patients with benign prostatic hyperplasia and 30 with prostate cancer at the Department of Urology, Medical University of Bialystok. The study protocol was approved by the Bioethics Committee of Medical University of Bialystok (APK.002.22.2024) and written informed consent to participate in the study was obtained from each person. The research material consisted of fragments of BPH and prostate cancer obtained during transurethral resection of the prostate or adenomectomy. Detailed histopathology data—such as specific Gleason scores or ISUP groupings—were not provided for research purposes and were not included in our database or this study. The collected material was immediately fixed in 10% buffered formalin and embedded in paraffin in a routine manner or placed in RNA‐later solution (AM7024 Thermo Fischer) and stored at −80°C. Paraffin blocks were cut into 4 μm‐thick sections and then stained with haematoxylin–eosin for general histological examination and processed for immunohistochemistry to detect ERK1/2, p38, β‐catenin, and E‐cadherin. The material stored in the RNA‐later solution was subjected to real‐time PCR to evaluate the expression of genes encoding ERK1/2, p38, β‐catenin, and E‐cadherin.

### 2.2 Immunohistochemistry

In the immunohistochemical study, the EnVision method was used, as previously described by Kasacka et al. [19]. Immunohistochemistry was performed, using an REAL EnVision Detection System, Peroxidase/DAB, Rabbit/Mouse detection kit (K5007; Dako Cytomation; Glostrup, Denmark). Immunostaining was performed by the following protocol: paraffin‐embedded sections were deparaffinised and hydrated in pure alcohols. For antigen retrieval, the sections were subjected to pretreatment in a pressure chamber heated for 1 min at 21 psi (one pound force per square inch (1 psi) equates to 6.895 kPa, the conversion factor has been provided by the United Kingdom National Physical Laboratory) at 125°C, using Target Retrieval Solution Citrate pH = 6.0 S 2369 (Dako Cytomation; Glostrup, Denmark) for ERK1/2, p38, β‐catenin and E‐cadherin. After cooling down to room temperature, the sections were incubated with Peroxidase Blocking Reagent S 2001 (Dako Cytomation; Glostrup, Denmark) for 10 min to block endogenous peroxidase activity. Subsequently sections were incubated with primary antibody for ERK1/2 (Rabbit polyclonal to p‐ERK1/2, 44‐680G Invitrogen), p38 (Rabbit polyclonal to p‐p38, 44‐684G Invitrogen), β‐catenin (Mouse Monoclonal to β‐catenin, ab32572, Abcam) and E‐cadherin (Rat Monoclonal to E‐cadherin, ab76055, Abcam). All antibodies were previously diluted in Antibody Diluent Background Reducing (S 3022 Dako Cytomation; Glostrup, Denmark) at a ratio of 1:50 for ERK1/2 and p38 antibodies, 1:2000 for β‐catenin and 1:500 for E‐cadherin antibody. Sections were incubated overnight at 4°C (incubation performed in a humid chamber). Then, incubation with a secondary antibody (conjugated to a horseradish peroxidase‐labelled polymer). Bound antibodies were visualised by 1‐min incubation with liquid 3,3′‐diaminobenzidine substrate chromogen. Finally, sections were counterstained with haematoxylin QS (H‐3404, Vector Laboratories; Burlingame, CA), mounted, covered and evaluated under a light microscope. Appropriate washing with Wash Buffer (S 3006 Dako Cytomation; Glostrup, Denmark) was performed between each step. In order to exclude non‐specific interactions between the tested material and the antibodies used, negative control reactions were performed in which the specific antibody of the detected antigens (ERK1/2, p38, β‐catenin and E‐cadherin) was replaced with normal rabbit serum (Vector Laboratories; Burlingame, California) in appropriate dilution. The results of these reactions were negative. The staining results were assessed using an Olympus BX43 light microscope (Olympus 114 Corp., Tokyo, Japan) coupled with an Olympus DP12 digital camera (Olympus 114 Corp., Tokyo, Japan) and documented.

### 2.3 Quantitative Analysis

Twelve sections of BPH and tumour were examined from each subject (three sections for each ERK1/2, p38, β‐catenin and E‐cadherin‐immunostaining). Five randomly selected microscopic fields (each field 0.785 mm2, 200× magnification (20× lens and 10× eyepiece)) from each prostate section were documented using an Olympus DP12 microscope camera. Each digital image was morphometrically evaluated using NIS Elements AR 3.10 Nikon microscopic image analysis software. In each analysed image, the intensity of the immunohistochemical reaction for all antibodies used in the study was measured on a grey scale from 0 to 256, where the value of a completely white or light pixel is 0, while the value of a completely black pixel is 256.

### 2.4 Real‐Time PCR

Samples of BPH and prostate cancer were taken from each patient and placed in an RNA‐later solution. Total RNA was isolated using the NucleoSpin RNA Isolation Kit (Machery‐Nagel). Quantification and quality control of total RNA were determined using a spectrophotometer—NanoDrop 2000 (ThermoScientific). An aliquot of 1 μg of total RNA was reverse transcribed into cDNA using the iScript Advanced cDNA Synthesis Kit for RT‐qPCR (BIO‐RAD). Synthesis of cDNA was performed in a final volume of 20 μL using a Thermal Cycler (Model SureCycler 8800, Aligent Technologies). For reverse transcription, the mixtures were incubated at 46°C for 20 min, then heated to 95°C for 1 min and finally cooled quickly at 4°C. Quantitative real‐time PCR reactions were performed using the Stratagene Mx3005P (Aligent Technologies) with the SsoAdvanced Universal SYBER Green Supermix (BIORAD). Specific primers for ERK1/2 (*MAPK3*, *MAPK1*), p38 (*MAPK14*), β‐catenin (*CTNNB1*), E‐cadherin (*CDH1*) and GAPDH (*GAPDH*) were designed by the BIORAD Company. The housekeeping gene GAPDH (*GAPDH*) was used as a reference gene for quantification. To determine the amounts of levels of test genes expression, standard curves were constructed for each gene separately with serially diluted PCR products. PCR products were obtained by cDNA amplification using specific primers as follows: *MAPK3* (qHsaCID0010939, BIO‐RAD), *MAPK1* (qHsaCED0042738, BIO‐RAD), *MAPK14* (qHsaCED0043417, BIO‐RAD), *CTNNB1* (qHsaCED0046518, BIO‐RAD), *CDH1* (qHsaCID0015365, BIO‐RAD) and *GAPDH* (qHsaCED0038674, BIO‐RAD). QRT‐PCR was carried out in a doublet in a final volume of 10 μL under the following conditions: 2 min polymerase activation at 95°C, 5 s denaturation at 95°C, 30 s annealing at 60°C for 40 cycles. PCR reactions were checked, including no‐RT‐controls, omitting templates, and a melting curve to ensure only one product was amplified.

### 2.5 miRNA Selection

To screen for interesting candidates, in silico prediction of putative miRNAs targeting BPH and prostate cancer‐related genes was performed. Our attention was focused on miRNAs related to the MAPK and Wnt/β‐catenin pathways because of their crucial importance in the development of urological cancers. For this purpose, screening of selected miRNAs was used using the NCBI mRNA database (NCBI mRNA DB: http://www.ncbi.nlm.nih.gov/). The following 2 miRNAs were selected for further analysis: 106a‐5p and 375‐3p.

### 2.6 Extraction of miRNA From Tissues

Extraction of miRNA from BPH and prostate cancer tissues was conducted using the miRNeasy Tissue/Cells Advanced Micro Kit (Qiagen, Copenhagen, Denmark, cat. no. 217684) according to the protocol prepared by the manufacturer. A section of 4 mm^3^ was removed from each tissue specimen and put into a 1.5 mL reaction tube along with 60 μL of lysis solution that contained 1% β‐mercaptoethanol. The tissue was homogenised with a disposable polypropylene pestle and placed in 700 μL of QIAzol Lysis Reagent (Qiagen, Copenhagen, Denmark, cat. no. 79306). Every step that followed was carried out in accordance with the manufacturer's instructions. 40 μL of RNase‐free water was used to elute the RNA (Qiagen, Copenhagen, Denmark, cat. no. 129112).

### 2.7 Quantification of RNA

A NanoDrop spectrophotometer (Thermo Fisher Scientific, Waltham, MA, USA) was used to measure the purity, concentration and contamination.

### 2.8 cDNA Synthesis

The research was carried out using the miRCURY LNA RT Kit (Qiagen, Copenhagen, Denmark, cat. no. 339340) to reverse transcription RNA. In the case of fresh frozen tissues of BPH and prostate cancer, the RNA concentration was adjusted to 5 ng/μL for each sample, and 2 μL was added to the 10 μL total reaction volume, which included 0.5 μL of UniSp6 RNA spike‐in.

### 2.9 Digital PCR (dPCR) Procedure

The dPCR reactions were performed using the miRCURY LNA miRNA PCR Assays kit (Qiagen, Copenhagen, Denmark, cat. no. 39306), a 96‐well plate format (Qiagen, Copenhagen, Denmark, cat. no. 250021), and the QIAcuity One nucleic acid detection instrument (Qiagen, Copenhagen, Denmark) with the specialised QIAcuity Software Suite software (Qiagen, Copenhagen, Denmark). Reaction mix was prepared according to the manufacturer's protocol, that is, 4 μL 3× EvaGreen PCR Master Mix (Qiagen, Copenhagen, Denmark, cat. no. 250111), 1.2 μL miRCURY LNA PCR Assay (10×) (Qiagen, Copenhagen, Denmark, cat. no. 339306), 3 μL cDNA template (Qiagen, Copenhagen, Denmark) and 3.8 μL of RNase‐free water (Qiagen, Copenhagen, Denmark, cat. no. 129112). Total reaction volume was 12 μL. 96‐well nanoplates with 8.500 partitions were used for the studies. The miRNAs used for the study were: 106a‐5p (Qiagen, Copenhagen, Denmark, cat. no. YP00204563) and 375‐3p (Qiagen, Copenhagen, Denmark, cat. no. YP00204362). The reaction mix was dispensed into the wells of a standard PCR plate, and the content of each well of the standard PCR plate was transferred to the QIAcuity Nanoplate (Qiagen, Copenhagen, Denmark, cat. no. 250021). The parameters for performing the dPCR reaction were consistent with the cycling conditions proposed by the manufacturer. This involved a 2 min PCR heat activation step at 95°C, 15 s denaturation at 95°C and 1 min annealing at 60°C, which constituted a 2‐step cycling (40 cycles), finally followed by cooling down for 5 min at 40°C. After the run was completed, the raw data were sent to the QIAcuity Software Suite (Qiagen, Copenhagen, Denmark), used for data analysis.

### 2.10 Statistical Analysis

All collected data were statistically analysed by means of the software computer package Statistica Version 13.3. Due to the lack of normality of the distribution of the obtained results, a non‐parametric Kruskal–Wallis test was performed to assess the statistical significance of differences between the BPH (epithelium and stroma) and cancer. Results on the intensity of the immunohistochemical reaction are presented as medians with minimum and maximum values. When the *p*‐value from theKruskal–Wallis test was less than 0.05 (*p* < 0.05), post hoc analysis using theMann–Whitney *U* test was conducted to compare specific pairs of groups (BPH epithelium vs. cancer, BPH stroma vs. cancer, BPH epithelium vs. BPH stroma) and to determine between which groups significant differences existed.

To assess the strength and direction of the interdependence between ERK1/2, p38, β‐catenin, and E‐cadherin in the epithelium of BPH, the stroma of BPH, and prostate cancer tissue, linear regression analysis was performed. This approach provided detailed insight into how the expression of one protein may influence the expression of another within a specific tissue microenvironment. In order to analyse the correlation between the tested proteins, statistically calculated *r* ^2^, the regression equation, and the correlation coefficient were used. The results of this analysis are shown as the *β* coefficient (this metric represents the percentage of the dependent variable's change for each unit of the independent variable's change), *r* ^2^ (shows the percentage of one variable that is responsible for the variability of the other), and the statistical significance (*p*). The relationship between two variables was acknowledged to be statistically significant at the value of the *β* coefficient for which *p* < 0.05.

In the QRT‐PCR method, the relative quantification of gene expression was determined by comparing Ct values using the ∆∆Ct method. All results were normalised to GAPDH. The miRNA results obtained for prostate cancer were normalised to those obtained for BPH.

## 3 Results

A total of 60 formalin‐fixed, paraffin‐embedded archival tissue blocks were included in the current study. The material included 30 prostate cancers and 30 benign prostatic hyperplasia (as comparative material) with an average age of 67 years. All surgical specimens were routinely histologically examined. For the purposes of our work, benign hyperplasia was separated from prostate cancer.

**FIGURE 1:**
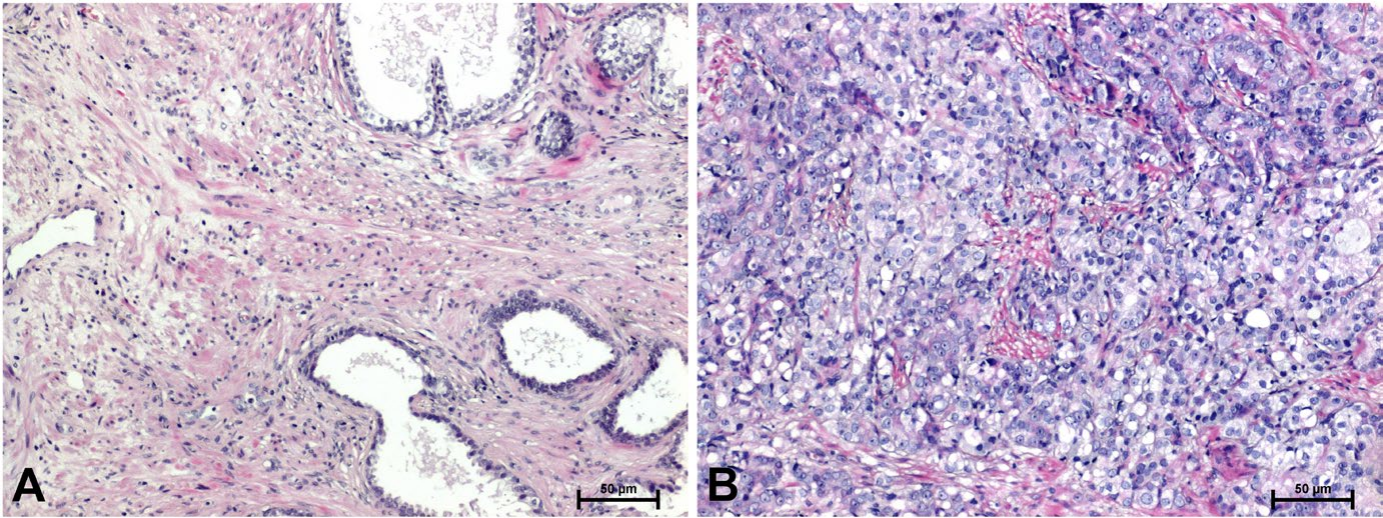
Hematoxylin and eosin staining in: (A) BPH and (B) prostate adenocarcinoma. Scale bars: 50 μm.

H + E staining revealed distinct histological differences between BPH and prostate cancer tissues. In BPH samples, the glandular structures appeared normally organised, and the connective tissue stroma did not show hyperplasia and features suggestive of stromal invasion (Figure 1A). In the case of prostate cancer, H + E staining allowed the observation of stromal invasion and cellular atypia (Figure 1B).

### 3.1 Immunohistochemical Evaluation

Immunohistochemical analysis showed significant weakening of immunoexpression for all antibodies used in the study.

**FIGURE 2:**
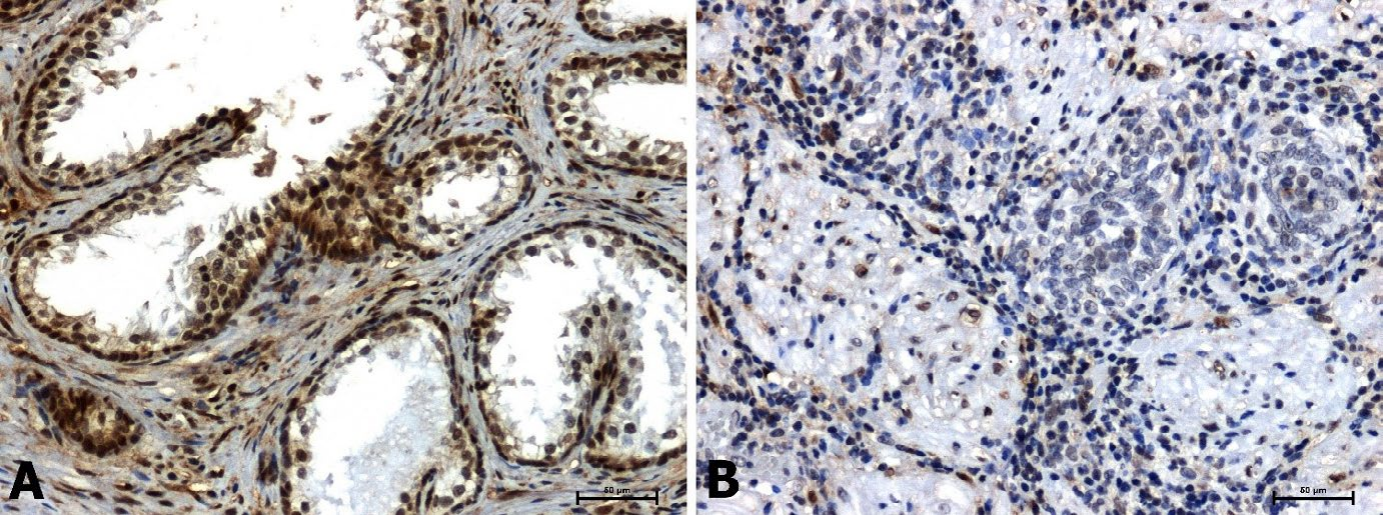
Immunoidentification of ERK1/2 in: (A) BPH and (B) prostate cancer. Scale bars: 50 μm.

**FIGURE 3:**
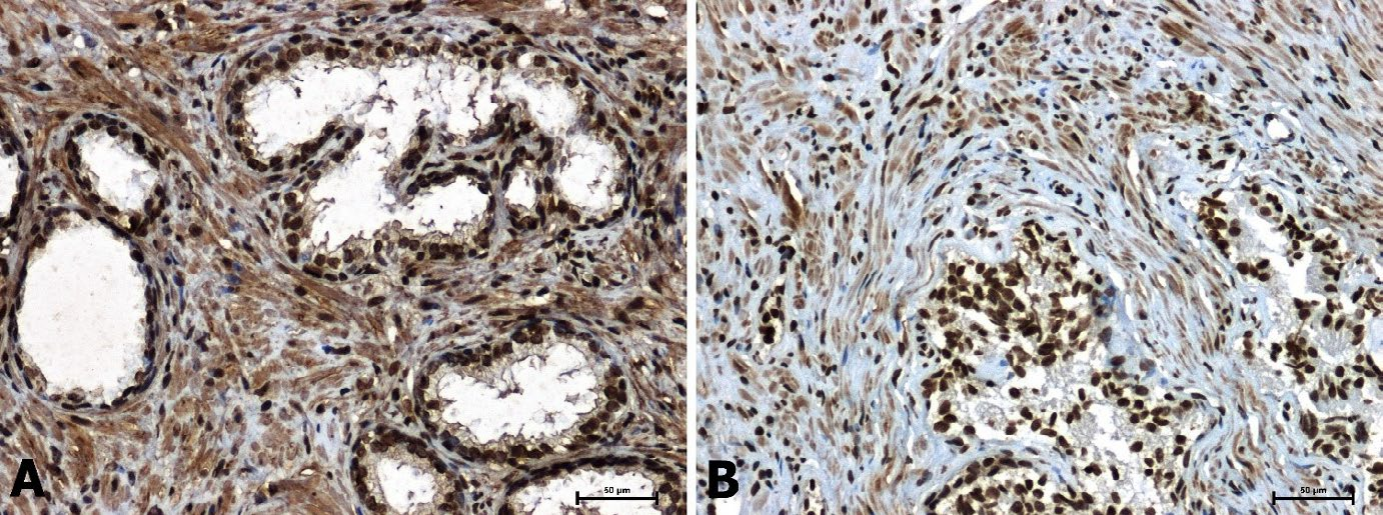
Immunolabelling of p38 in: (A) BPH and (B) prostate adenocarcinoma. Scale bars: 50 μm.

**FIGURE 4:**
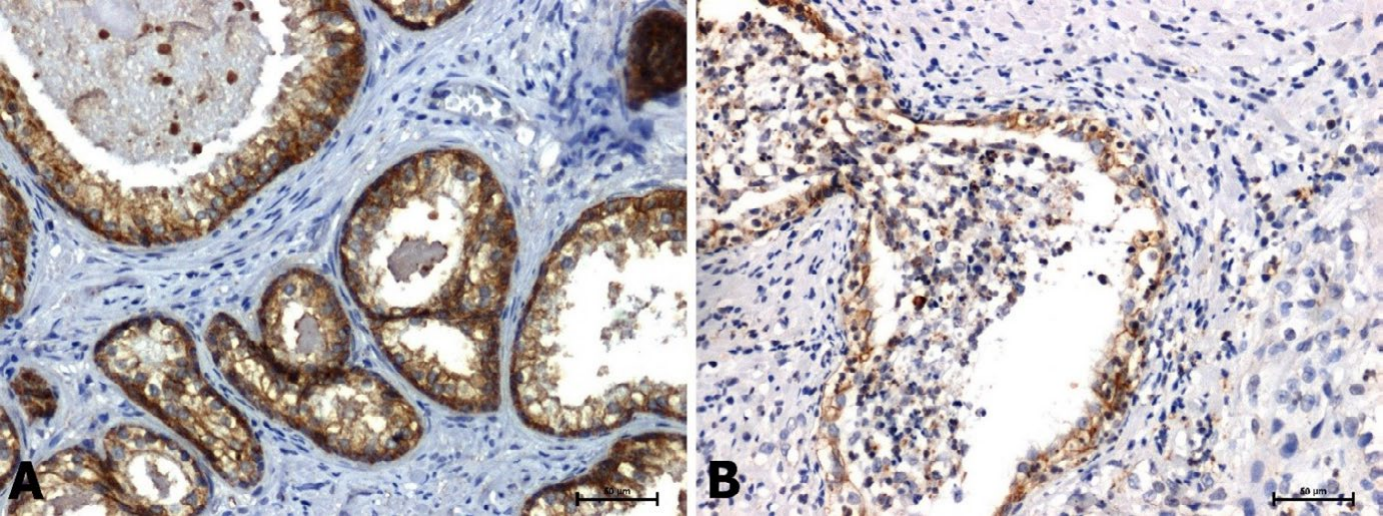
Immunodetection of β‐catenin in: (A) BPH and (B) prostate cancer. Scale bars: 50 μm.

**FIGURE 5:**
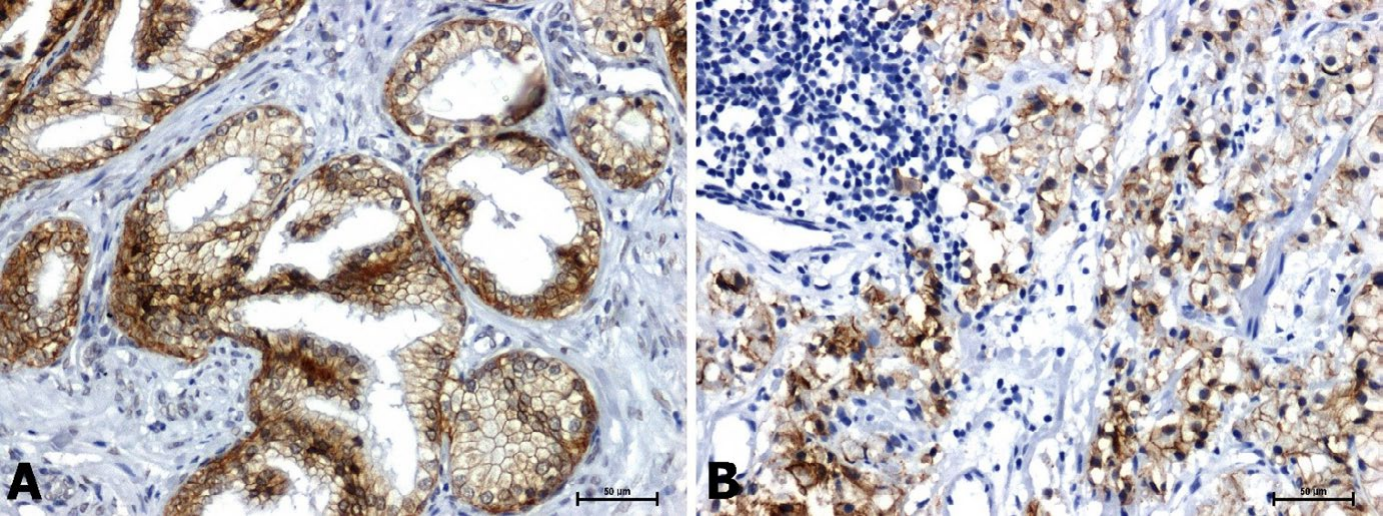
Immunoreactivity of E‐cadherin in: (A) BPH and (B) prostate adenocarcinoma. Scale bars: 50 μm.

The results of the assessment of selected tissue antigens (ERK1/2, p38, β‐catenin, E‐cadherin) are shown in Figures 2, 3, 4, 5 and in Table 1.

**TABLE 1:** The intensity of immunoreaction determining ERK1/2, p38, β‐catenin, and E‐cadherin in BPH and prostate cancer.

*Intensity of immunohistochemical reaction in benign prostatic hyperplasia and prostate cancer*				
Scale from 0 (white pixel) to 256 (black pixel)				
	ERK1/2	p38	β‐catenin	E‐cadherin
BPH—epithelium	134.1 99.32–166.1	191.5 170.7–206.0	137.3 80.70–160.2	76.83 64.75–107.9
BPH—stroma	115.5 89.30–129.1***	135.1 118.7–149.7***	49.61 32.37–63.86***	36.69 22.15–72.23***
Prostate cancer	38.69 7.620–75.01*,**	59.73 32.36–75.97*,**	49.31 23.36–81.09*	64.83 33.54–74.66*

*Note:* Data are shown as a median and minimum and maximum values for ERK1/2, p38, β‐catenin and E‐cadherin in epithelium and stroma of BPH and prostate cancer.

* *p* < 0.05—prostate cancer vs. BPH epithelium.

** *p* < 0.05—prostate cancer vs. BPH stroma.

*** *p* < 0.05—BPH stroma vs. BPH epithelium.

In benign prostatic hyperplasia, intense ERK1/2 immunoreactivity was observed both in glandular epithelial cells and in the connective tissue stroma (Figure 2A). The immunoreactivity of ERK1/2 in all prostate cancer tissues was very low or negative (Figure 2B).

In non‐neoplastic tissues, a strong p38 reaction was found in both glandular epithelial cells and connective tissue stroma (Figure 3A). In tumour tissues, p38 immunoreactivity was significantly attenuated compared with BPH (Figure 3B).

Similarly, strong β‐catenin immunoreactivity was found in BPH (Figure 4A) and a marked attenuation of the reaction in prostate cancer (Figure 4B). In both tissues examined, the membrane localization of β‐catenin was demonstrated.

The results of the reaction using the anti‐E‐cadherin antibody were similar. Marked immunoexpression in membrane localization in non‐tumour tissues (Figure 5A) and significant attenuation of the reaction in tumour tissues (Figure 5).

The measurement of the intensity of the immunohistochemical reaction using computer image analysis, confirmed by statistical evaluation, revealed lower immunoreactivity of ERK1/2, p38, β‐catenin, and E‐cadherin in prostate cancer compared to BPH (epithelium and stroma). The presented results were statistically significant, with the exception of the differences between prostate cancer and BPH stroma for β‐catenin and E‐cadherin expression. Furthermore, differences in the immunostaining intensity of the analysed proteins were compared within the control group, specifically between BPH stroma and BPH epithelium. This analysis demonstrated that in BPH stroma, the immunoreactivity of all analysed proteins was significantly lower compared to BPH epithelium (Table 1).

### 3.2 Real‐Time PCR

**FIGURE 6:**
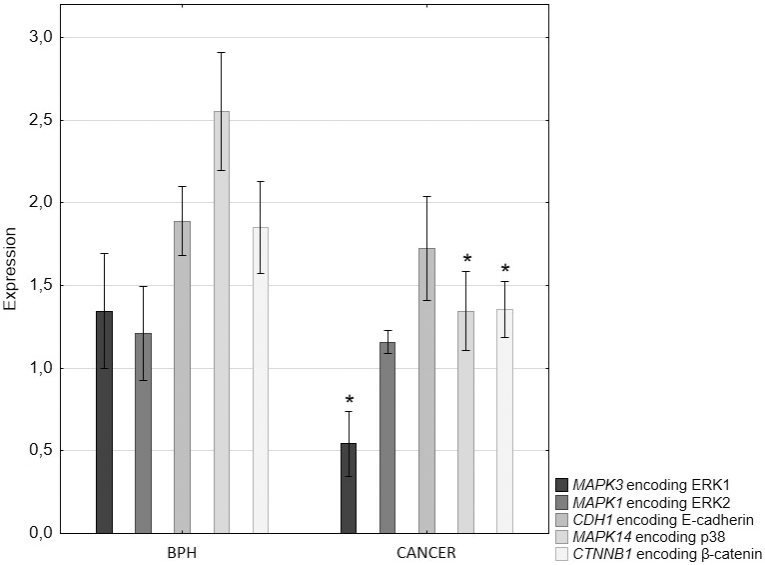
Expression of genes encoding ERK1/2 (*MAPK3/MAPK1*), p38 (*MAPK14*), β‐catenin (*CTNNB1*) and E‐cadherin (*CDH1*) in BPH and prostate cancer. *p < 0.05—prostate cancer vs. BPH.

QRT‐PCR analysis showed significantly lower expression of the genes: *MAPK3* encoding ERK1 and *MAPK1* encoding ERK2, p38, β‐catenin and E‐cadherin in prostate adenocarcinoma compared to BPH tissues (Figure 6). Statistically significant differences were found in the gene encoding ERK1, p38 and β‐catenin (Figure 6).

Statistical analysis showed co‐expression between all parameters we examined, these results were marked with an asterisk *(*p* < 0.05). As for the relationships between the tested proteins, in the epithelium and stroma of BPH patients and patients with prostate cancer, mutually positive relationships were found between each protein tested. The results of the correlation between the tested proteins, divided into BPH and prostate cancer are presented in the Table 2 and Table 3.

**TABLE 2:** Correlation analysis between the tested proteins in benign prostatic hyperplasia.

Benign prostatic hyperplasia—Epithelium				
ERK1/2	p38	β‐catenin	E‐cadherin	
—	*β* = +0.6305 *p* = 0.00001* *r* ^2^ = 0.9313	*β* = +0.9908 *p* = 0.0002* *r* ^2^ = 0.8370	*β* = +0.7553 *p* = 0.00008* *r* ^2^ = 0.8711	ERK1/2
	—	*β* = +1.3932 *p* = 0.0023* *r* ^2^ = 0.7063	*β* = +1.14 *p* = 0.0002* *r* ^2^ = 0.8470	p38
		—	*β* = +0.5509 *p* = 0.0150* *r* ^2^ = 0.5435	β‐catenin
			—	E‐cadherin

Benign prostatic hyperplasia—Stroma				
ERK1/2	p38	β‐catenin	E‐cadherin	
—	*β* = +0.806 *p* = 0.00001* *r* ^2^ = 0.9309	*β* = +0.8087 *p* = 0.00000* *r* ^2^ = 0.9444	*β* = +0.982 *p* = 0.0019* *r* ^2^ = 0.7187	ERK1/2
	—	*β* = +0.9692 *p* = 0.00000 * *r* ^2^ = 0.9469	*β* = +1.2198 *p* = 0.0008* *r* ^2^ = 0.7741	p38
		—	*β* = +1.2669 *p* = 0.0003* *r* ^2^ = 0.8289	β‐catenin
			—	E‐cadherin

*Note:* The results of the analysis of regression are presented as the *β* coefficient, *r* ^2^, and the level of statistical significance (*p*, where *p < 0.05).

**TABLE 3:** Correlation analysis between the tested proteins in prostate cancer.

Prostate cancer				
ERK1/2	p38	β‐catenin	E‐cadherin	
—	*β* = +0.5249 *p* = 0.00009* *r* ^2^ = 0.9017	*β* = +0.857 *p* = 0.0016* *r* ^2^ = 0.7783	*β* = +0.6339 *p* = 0.0239* *r* ^2^ = 0.5411	ERK1/2
	—	β = +1.2797 *p* = 0.00007* *r* ^2^ = 0.8735	*β* = +1.16 *p* = 0.0002* *r* ^2^ = 0.8382	p38
		—	*β* = +0.8303 *p* = 0.0004* *r* ^2^ = 0.8053	β‐catenin
			—	E‐cadherin

*Note:* The results of the analysis of regression are presented as the *β* coefficient, *r* ^2^, and the level of statistical significance (*p*, where *p < 0.05).

**FIGURE 7:**
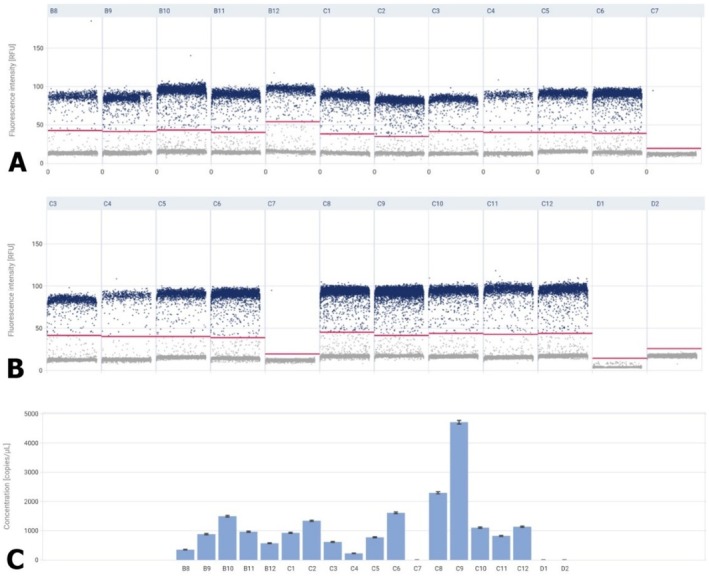
Expression analysis of miRNA 106a‐5p. In panels A and B of the Figure 7, the X‐axis shows single samples and the Y‐axis shows fluorescence intensity of miRNA 106a‐5p. Each point represents the signal from a single partition, with higher values typically representing positive results (presumptive amplification) and lower values typically representing negative results (background signal). Panel C presents the concentration diagram (copies/μL), which shows the concentrations of samples in a column chart.

**FIGURE 8:**
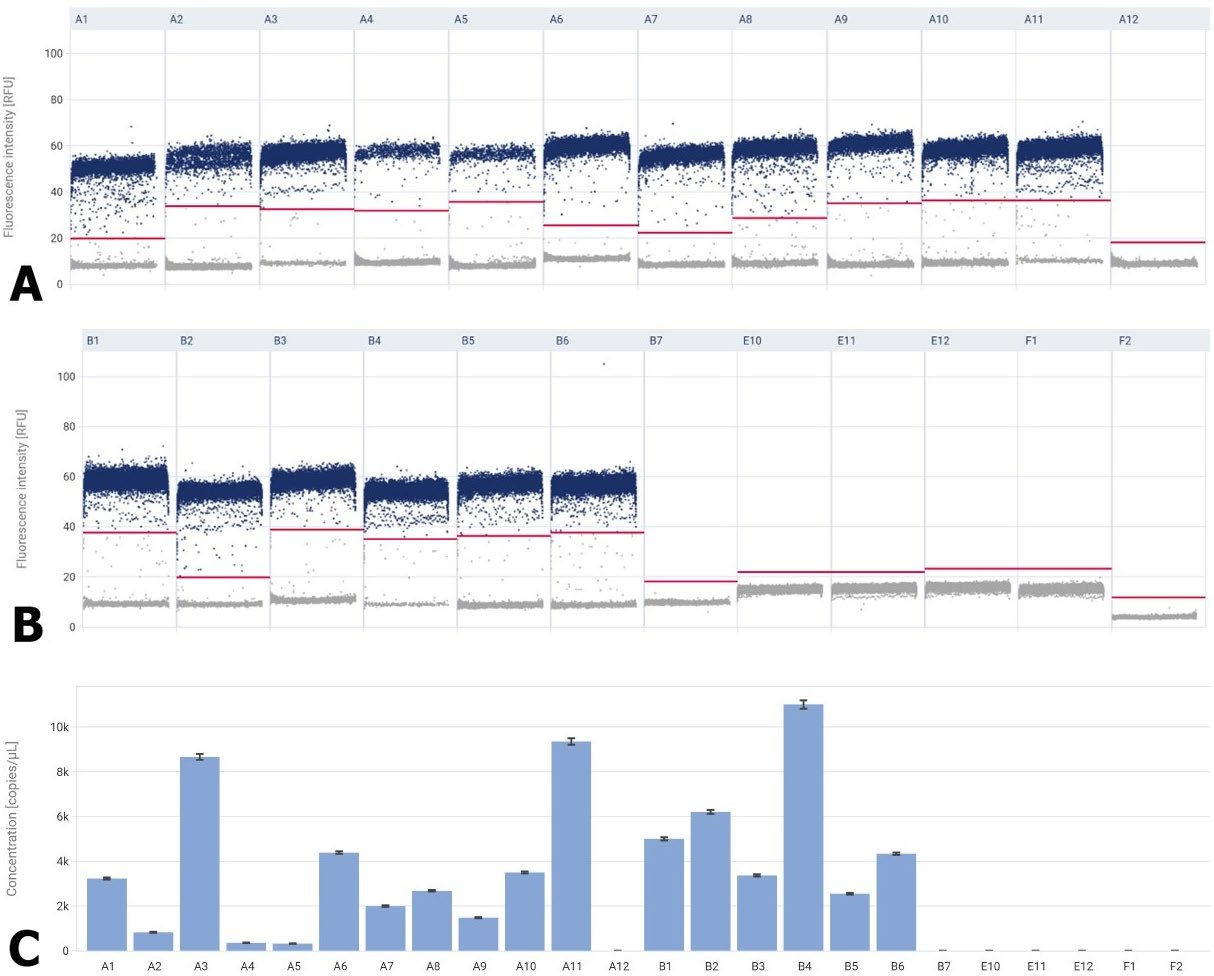
Expression analysis of miRNA 375‐3p. In panels A and B of Figure 8 the X‐axis shows single samples, and the Y‐axis shows fluorescence intensity of miRNA 375‐3p. Panel C presents the concentration diagram (copies/μL), which shows the concentrations in the total volume of the samples.

**FIGURE 9:**
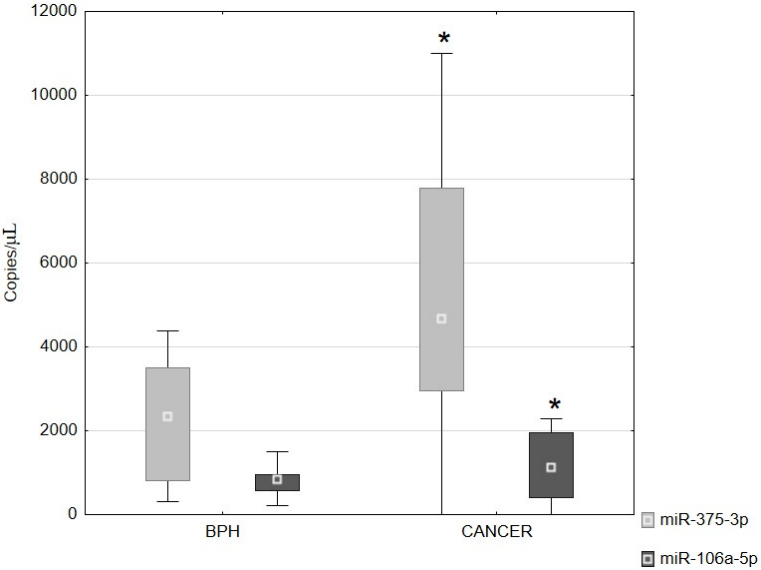
Expression levels of miRNAs in BPH and prostate cancer. Data are shown as a median (Kruskal–Wallis test). *p < 0.05—prostate cancer vs. BPH.

A sample containing the reaction mixture with no template control, that is, NTC, was used as a negative control for the studies. In the case of both tested miRNAs, the NTC was 0. The most important parameter in miRNA testing is the ‘Concentration’ expressed in copies/μL. The tested miRNAs showed changes in fluorescence intensity between benign prostatic hyperplasia and prostate cancer. In the case of miR‐106a‐5p and miR‐375‐3p, the fluorescence intensity of these miRNAs was statistically significantly higher in cancer compared to BPH (Figures 7, 8, 9). As shown in Figure 8, in the prostate cancer tissue concentration level of miRNA106a‐5p was 1118.8 copies/μL (median), while miRNA 375‐3p showed concentrations of 4667.0 copies/μL (median).

The miRNA results obtained for prostate cancer were normalised to those obtained for BPH. For miR‐375‐3p the result was 1905 and for miR‐106a‐5p 1796.

## 4 Discussion

Prostate adenocarcinoma is the most frequently diagnosed malignancy and the most common cause of death due to the cancer in men. Therefore, this cancer has become one of the main challenges for scientists and medics. A detailed understanding of the molecular mechanisms and the identification of new biomarkers are important for the early diagnosis and effective treatment strategy of prostate cancer. Great hope in the development of new prognostic and therapeutic methods in oncology is associated with the latest discovery, a small non‐coding miRNA molecule. Research to date has shown that miRNA molecules play a key role in the carcinogenesis processes of prostate cancer [20, 21]. By influencing androgen receptor overexpression, cell adhesion, resistance to apoptosis, and epithelial‐mesenchymal transition, miRNAs play important roles in cancer growth and development. The miRNA we studied may potentially demonstrate utility as a biomarker in prostate cancer.

Activation of the MAPK pathway, in particular ERK kinase, or mutations in the RAS–RAF–MEK–ERK signalling pathway are considered to be critical events in tumourigenesis of various tissue types [14]. In this study, we demonstrated lower immunoreactivity and expression of ERK1/2 in prostate cancer compared with benign prostatic hyperplasia.

Research by Shin et al. [14], showed that miR‐106a affects the activity of MAPK kinases in bladder cancer cells by reducing ERK phosphorylation and increasing p38 phosphorylation.

The results of studies by other authors and our own research may indicate that cancer cells may be dependent on growth stimuli caused by the activation of ERK kinase [14, 22]. Apart from that, in studies conducted by Wu [22], it was observed that in addition to the inhibition of p‐ERK1/2, activation of other stress‐activated MAP kinases, that is, p38 and JNK, was demonstrated in miR‐106a transfectants. These findings may suggest that miR‐106a inhibits prostate cancer cell proliferation by suppressing ERK or other elements of the MAPK pathway.

ERK kinase is involved in the regulation of cell growth and differentiation, while p38 kinase is mainly responsible for maintaining inflammation and cell apoptosis [23]. The present study showed significantly lower p38 immunoreactivity and expression in prostate adenocarcinoma compared with BPH.

A team of researchers led by Park [24] also demonstrated lower p38 expression and inactivation of p38 kinase in prostate cancer cell lines. MAP kinases, due to their phosphorylation, influence the survival, apoptosis and differentiation of cancer cells. The regulation of MAP kinases may depend on the expression of specific genes [24]. Perhaps suppression of one of the genes we tested causes dephosphorylation of p38 kinase. Non‐coding miRNA molecules serve as transcriptional and post‐transcriptional regulators of gene expression by forming base pairs with messenger RNA (mRNA).

Cheung et al. [25] in experimental studies showed that p38 inhibition may prolong the survival of tumour‐bearing animals and reduce the development of prostate cancer. The participation of p38 kinase in the process of carcinogenesis is also confirmed by the results of research by Royuela et al. [26] and Yao et al. [27]. In experimental studies, Zhu et al. [23] showed that the use of a p38 kinase inhibitor and lowering the level of its phosphorylated form results in inhibition of cell proliferation in prostate cancer.

According to literature data, disruption of the Wnt/β‐catenin signalling pathway positively correlates with the progression of prostate cancer [28]. Our studies revealed statistically significantly lower immunoreactivity and expression of the gene encoding β‐catenin.

Results inconsistent with our study were obtained by Aldahl et al. [28], who showed higher expression of β‐catenin in prostate cancer in transgenic mice. Differences in the results of these studies may indicate different mechanisms influencing the β‐catenin pathway in the development of prostate cancer. The authors of these studies used genetically modified animals, which are only an experimental model reflecting human diseases. Furthermore, physiological and metabolic differences between humans and animals must be taken into account. Despite many similarities in the development of cancer in animals and humans, there are also many differences, for example, related to metabolism, the formation of free radicals, or the neutralisation of carcinogens.

E‐cadherins are key proteins in maintaining functional adhesion junctions in epithelial tissue and tissue integrity [29]. The present study showed lower E‐cadherin levels in prostate cancer compared to BPH tissues. The present study showed lower E‐cadherin levels in prostate cancer compared to BPH tissues. This may be related to the cleavage of E‐cadherin into smaller molecules and a reduction in cells showing signs of apoptosis. It can be assumed that the lower expression of this protein in prostate cancer depends on the mechanisms of cell death.

Literature data indicate that reduced levels of E‐cadherin expression induce invasion and metastasis of cancer cells [29, 30]. Consistent with our results, a reduction in the level of E‐cadherin in prostate cancer cells was obtained by Varisli and Tolan [29] while indicating reduced cell cohesion in primary tumours and the spread of cancer cells to the bone.

Previous studies have shown the involvement of miR‐106 in the development of many cancers, including prostate cancer [1]. The suppressor activity of miR‐375 has been proven mainly in gastrointestinal cancers for example, stomach, pancreas, or liver cancer [15]. It has also been shown that miR‐375 can inhibit the growth of cancer cells due to cell arrest in the G0 phase [31].

In our studies, we demonstrated a statistically significant higher fluorescence intensity of miR‐106a‐5p and miR‐375‐3p in cancer compared to BPH.

Higher miR‐106a‐5p expression in prostate cancer compared to normal tissue was also shown by studies by Lin et al. [32]. These authors demonstrated that inhibition of this miRNA promotes the invasion and migration of prostate cancer cells.

MiRNA is characterised by high stability, and remains resistant to RNase, temperature, and pH of plasma, serum, saliva, freshly frozen tissues, and paraffin blocks for a long period of time. In the aspect of clinical diagnostics, the above features determine miRNA as excellent biomarkers [9]. An easy and non‐invasive way to obtain miRNA circulating in the blood may prove to be an extremely important element in medicine, especially oncology. However, the role of miRNAs as biomarkers requires further detailed research.

As shown in the study by Bidarra et al. [21], miR‐375‐3p in the plasma was not overexpressed in patients with early metastatic changes of prostate cancer; however, its expression increased along the progression of this tumour and significantly correlated with lower metastasis‐free survival, which can suggest the usefulness of miR‐375‐3p as the prognostic biomarker of disease progression.

Abramovic et al. [33] using droplet digital PCR showed statistically significantly higher expression of miR‐375‐3p in the plasma of men with prostate cancer compared to patients with BPH. Results consistent with ours were also obtained by other authors who compared the expression of miR‐375‐3p in blood plasma of patients with prostate cancer compared to patients with BPH [34, 35, 36]. According to literature sources, miR‐375‐3p expression is also strongly correlated with the progression of prostate cancer [37].

The results of the study by Wei et al. [15] showed that in miR‐375 over‐expressing cells, ERK kinase was down‐regulated and cell proliferation was inhibited. These authors indicate that miR‐375 acts as a tumour repressor gene to reduce cell proliferation through the ERK kinase pathway in colorectal cancer.

It is possible that thanks to miRNA 106a‐5p and 375‐3p, the proteins we studied belonging to the MAPK and Wnt/β‐catenin pathways play a protective suppressor role in prostate cancer cells. Moreover, we compared the differences in the intensity of immunostaining of ERK1/2, p38, β‐catenin, and E‐cadherin in the control group—between BPH stroma and BPH epithelium and—showed that lower immunoreactivity of MAPK and Wnt/β‐catenin pathway proteins in BPH stroma compared to epithelium may indicate differential activity of these pathways in individual tissue regions. This may suggest that signalling processes associated with proliferation and differentiation are more intense in epithelial cells and that these differences may reflect different biological functions of stroma and epithelium in the pathogenesis of BPH. Additionally, the results of the linear regression analysis showed that an increase in the expression of one of the analysed proteins was associated with a simultaneous increase in the expression of the others within the given tissue microenvironment. This may suggest a coordinated activation of components of the MAPK and Wnt/β‐catenin pathways in BPH and prostate cancer tissue.

The aim of our further research will be to investigate other miRNA in the tissues and blood of more patients with BPH and prostate cancer, which will allow on to better understand differences between benign hyperplasia and cancer.

The limitation of our study is the lack of evaluation of selected miRNAs in the circulating blood of patients; however, we only had fresh frozen tissue material collected during surgery. In our studies, we related the results regarding prostate cancer to BPH due to the lack of healthy tissue, which could not be obtained from the study patients. We are aware that the results in healthy tissues may differ from those observed in BPH, and we will try to check how much if possible?

## 5 Conclusions

In conclusion, the results of the obtained studies will provide novel data on the relationship between miRNAs 106a‐5p and 375‐3p with the proteins ERK1/2, p38, β‐catenin and E‐cadherin in prostate cancer compared to BPH. Confirmed by further research, they may have great clinical value in the future as reference points in the design of biomarkers enabling the differentiation of cancer from benign prostatic hyperplasia.

Further studies of prostate biomarkers are needed to assess the risk of disease recurrence and progression and to differentiate low‐risk patients who require less aggressive treatment from those who should receive more invasive therapy.

## Author Contributions

**Magdalena Smereczańska:** conceptualization (equal), investigation (supporting), methodology (supporting), resources (lead), writing – original draft (lead). **Natalia Domian:** methodology (supporting), resources (supporting), validation (lead). **Grzegorz Młynarczyk:** formal analysis (lead), investigation (supporting), methodology (supporting), validation (equal), visualization (lead). **Irena Kasacka:** conceptualization (equal), investigation (supporting), methodology (supporting), resources (supporting), supervision (lead), writing – review and editing (lead).

## Ethics Statement

This study was approved by the Bioethics Committee of the Medical University of Bialystok. The code for our study is APK.002.22.2024.

## Consent

Informed consent was obtained from all subjects involved in the study.

## Conflicts of Interest

The authors declare no conflicts of interest.

## Data Availability

Data from this study are available from the corresponding author upon reasonable request.
